# Research on the evaporation pressure calculation model of LNG tank container

**DOI:** 10.1371/journal.pone.0314635

**Published:** 2025-05-12

**Authors:** Zhidong Yu, Bao Li

**Affiliations:** 1 Zhejiang Technical Institute of Economics, Hangzhou, Zhejiang, China; 2 Zhejiang Institute of Mechanical and Electrical Engineering, Hangzhou, Zhejiang, China; University of Shanghai for Science and Technology, CHINA

## Abstract

The changes in pressure, temperature, and filling rate inside LNG tank container are related to non-destructive storage time. In order to solve these problems, it was necessary to understand the distribution of temperature, pressure and evaporation in the LNG storage tank during transportation. The study on the relationship between the initial filling rate and safe storage time is still rare. Based on the principle of mass conservation and energy conservation, the pressure calculation model of the horizontal low-temperature LNG tank was established and verified by an experiment. A study was conducted on the LNG tank container in Golmud-Lhasa, and experimental data such as pressure changes in LNG tank containers during transportation were obtained. The storage calculation results obtained by the model were compared with the experimental data. The research results show that the simulated calculated values agree well with the experimental values, there was an “optimal initial filling rate” in the LNG tank. When the initial filling rate was less than the optimal initial filling rate, the safe storage time of the tank increases with the increase of the initial filling rate. When the initial filling rate was greater than the optimal initial filling rate, the safe storage time of the tank decreases with the increase of the initial filling rate.

The transportation of hazardous goods by rail is characterized by high technical requirements, significant safety risks, and complex properties. In recent years, China’s rail transport safety services for hazardous goods have improved, but issues remain such as long transit times, high risk factors, and weak safety prevention capabilities. Due to the ultra-low temperature properties of Liquefied Natural Gas (LNG), there is a significant temperature difference between the LNG inside the tank and the external environment during storage and transportation. This causes the LNG to continuously absorb heat from the environment, leading to the evaporation of the low-temperature liquid into gas and increasing the vapor pressure inside the tank, thereby affecting the safety of LNG tank container transportation. In the event of a leak during transportation, LNG rapidly absorbs heat and vaporizes, causing frost formation on the surface of the storage equipment. This leads to the failure of the tank’s material, such as reduced strength and hardness, contraction in the cold, or brittle fractures, resulting in pipeline or tank ruptures, which can further cause cold burns to transportation personnel, leading to confusion or asphyxiation over time. Under normal temperature and pressure, LNG vaporizes and spreads rapidly, and when encountering an open flame, it can easily cause fires or explosions. Therefore, to ensure the safe transportation of LNG tank containers, it is necessary to thoroughly study the temperature and pressure changes during the transportation process [1–5]. These researches mainly focus on the physical evolution laws of LNG, and the research results in materials science also have reference significance [6–8]. Liang and Zhen [9] analyzed the safety of LNG tank container railway transportation, summarized typical hazards and countermeasures, and pointed out that railway transportation of LNG tank containers is generally longer than road transportation requiring theoretical and practical measures to ensure sufficient safe storage time. Chen et. al. [10] proposed a revised model after analyzing the homogeneous surface evaporation model, thermal analysis model, and Russian model. This model did not consider the temperature distribution imbalance in the LNG liquid phase. Li et. al. [11] conducted research on LNG stratification and rollover phenomena, dividing the rollover process into four stages: fixed interface stage, upper layer carrying the lower layer, central jet penetrating the boundary layer, and rollover. The simulation results explained the accelerated decline of the stratified interface, but the research findings could not visually demonstrate the actual process.

The above researches lack exploration of the relationship between the initial filling rate and safe storage time. Additionally, many existing mathematical models lack experimental data validation. This article establishes a multi-parameter model and verifies its accuracy through experiments.

## 1 Insulation structure of LNG tank container

The insulation layer of the tank container controls the transfer of heat from the surrounding environment to the interior of the equipment, ensuring the normal transportation of LNG and reducing evaporation and vaporization. For the insulation layer of the LNG tank container, heat is mainly transferred through conduction and convection. Due to the large temperature variation inside and outside the LNG tank container during transportation, wide-range temperature fluctuations can easily cause damage to the insulation structure of the LNG tank container, thereby leading to the risk of LNG leakage.

**Fig 1:**
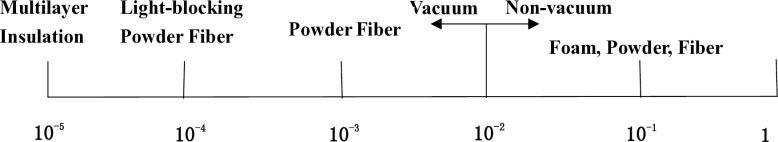
Thermal conductivity of different insulation types (W/m K).

The types of low-temperature insulation layers mainly include bulk insulation, high vacuum insulation, vacuum powder insulation, vacuum fiber insulation, high vacuum multilayer insulation, and high vacuum multi-shield insulation (Fig 1). From the effective thermal conductivity of different insulation types, it is observed that high vacuum multilayer insulation has better performance; thus, “high vacuum” multilayer insulation is adopted. The thermal resistance in multilayer insulation includes solid conduction thermal resistance, radiation thermal resistance, and residual gas conduction thermal resistance. Due to the complexity of the structure and the anisotropic behavior characteristics of the insulation, the heat transfer mechanism of high vacuum multilayer insulation is very complex. In engineering, the overall apparent thermal conductivity is used to characterize the thermal conductivity performance of multilayer insulation.

The heat transfer situation between the tank wall and the liquid in low-temperature containers is also very complex. It includes heat transfer by gas molecules, radiation heat transfer in the insulation space and at the pipe openings, heat transfer through the insulation material, and heat transfer of mechanical components. In engineering calculations, the insulation layer is assumed to be a one-dimensional temperature field; the temperature gradient exists only in the vertical direction; the thermal resistance of the inner tank and outer shell of the LNG tank container can be ignored in the calculation process; and the fluid inside the LNG tank container is in a saturated homogeneous state.

## 2 Evaporation rate control equation for LNG tank containers

**Fig 2:**
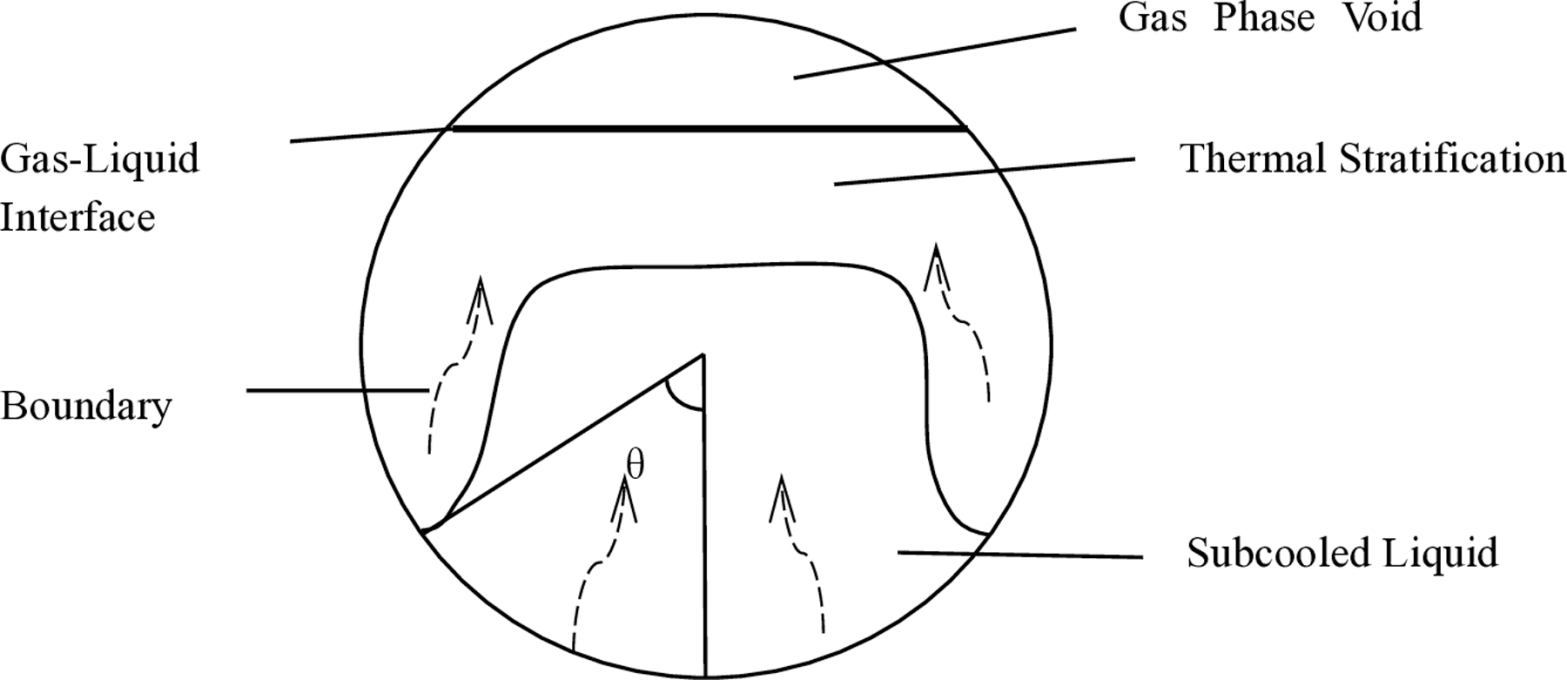
Heat transfer boundary layer in horizontal tank.

For LNG tank containers, external environmental heat is transferred into the tank through the tank wall. The low-temperature liquid, once heated, forms a natural convection boundary layer at the tank wall. Many researchers have studied the fluid boundary layer formed on such concave surfaces using boundary layer theory [12]. The boundary layer of a horizontally placed storage tank is shown in Fig 2, where the angle θ plays a very important role in the study of the concave surface boundary layer, with a boundary layer angle of 63.5°. Studies have shown that due to the existence of the boundary layer, thermal stratification forms near the gas-liquid interface in the low-temperature liquid, with the temperature of this fluid layer being significantly higher than that of the supercooled main fluid. After absorbing heat from the wall, the fluid boundary layer ultimately flows into the thermal stratification zone [13–16].

Most LNG tank containers are of the vacuum insulation type, with a structure composed of an outer cylinder wall, an insulated vacuum insulation layer, an inner tank, and supporting components. For the sake of calculation convenience, the following assumptions are made:

(1) Lateral heat conduction of the insulated vacuum insulation layer is neglected, and it is assumed that the heat transfer of the entire LNG tank container is one-dimensional, perpendicular to the insulation layer. If multiple insulation layers are present, they can be considered as a single insulation layer composed of multiple thermal resistances in the calculation.
(2) The thermal resistance of the external and internal metal cylinders is neglected, as well as the internal energy changes caused by heat transfer through these metal cylinders. The thickness of the temperature gradient on the gas-liquid interface is also neglected, and all the heat absorbed by the LNG tank container from the environment is assumed to be absorbed by the gas and liquid phases.
(3) The heat leakage of the supporting components of the LNG tank container is neglected.

The evaporation rate is an important parameter for measuring the sealing performance of LNG storage tanks and is also a crucial parameter for design. It is generally assumed in engineering that the temperatures of the gas and liquid phases are always consistent, and the liquid phase is always in a saturated state. However, the evaporation degrees of each component vary, leading to different mole fractions of each component in the gas and liquid phases. As the pressure inside the tank increases, the boiling point of LNG also changes. Therefore, to meet design requirements, the evaporation rate of the storage tank must be calculated and determined.

## 3 Heat leakage calculation for LNG tank containers

Using the three-phase model, all the heat transferred from the environment is absorbed by the gas and liquid phases inside the tank. The calculations are as follows:

The convective heat transfer between the outer wall of the storage tank and the environment, i.e., the convective heat transfer between the air and the outer wall, is expressed as follows:


$$Q_{0}=\alpha_{hw}F_{0}\left(T_{hw}-T_{0}\right)$$ (1)

where *α*_*hw*_ is the convective heat transfer coefficient of air, *F*_*0*_ is the heat transfer area of the outer wall of the storage tank, *T*_*hw*_ is the ambient temperature, and *T*_*0*_ is the temperature of the outer wall of the storage tank.

The heat transfer through the insulation layer of the tank wall, after neglecting the energy losses of the inner and outer cylinders, can be approximated as follows:


$$Q_{e}=\lambda_{e}\frac{F_{b}}{\sigma }\left(T_{0}-T_{b}\right)$$ (2)

where *λ*_*e*_ is the thermal conductivity of the tank wall, *F*_*b*_ is the heat transfer area of the tank wall, and *T*_*b*_ is the temperature of the inner wall of the storage tank.

The convective heat transfer between the liquid phase inside the tank and the inner wall is expressed as follows:


$$Q_{1}=\alpha_{bl}F_{bl}\left(T_{b}-T_{l}\right)$$ (3)

where *α*_*bl*_ is the convective heat transfer coefficient of the liquid inside the storage tank, *F*_*bl*_ is the heat transfer area between the inner wall of the storage tank and the liquid, and *T*_*l*_ is the temperature of the liquid inside the storage tank.

The convective heat transfer between the gas phase inside the tank and the inner wall is expressed as follows:


$$Q_{g}=\alpha_{bg}F_{bg}\left(T_{b}-T_{g}\right)$$ (4)

where *α*_*bg*_ is the convective heat transfer coefficient of the gas inside the storage tank, *F*_*bg*_ is the heat transfer area between the inner wall of the storage tank and the gas, and *T*_*g*_ is the temperature of the gas inside the storage tank.

The total heat leakage of the storage tank is expressed as follows:


$$Q=Q_{e}=Q_{l}+Q_{g}$$ (5)

The total heat leakage, after rearranging the above expressions, is represented as follows:


$$Q=\frac{\alpha_{bl}\alpha_{hw}\lambda_{e}F_{bl}F_{w}F_{b}\left(T_{hw}-T_{l}\right)+\alpha_{bg}\alpha_{hw}\lambda_{e}F_{bg}F_{w}F_{b}\left(T_{hw}-T_{g}\right)}{\lambda_{e}\alpha_{hw}F_{w}F_{b}+\alpha_{bl}F_{bl}\left(\sigma \alpha_{h}F_{w}+\lambda_{e}F_{b}\right)+\alpha_{bg}F_{bg}\left(\sigma \alpha_{h}F_{w}+\lambda_{e}F_{b}\right)}$$ (6)

## 4 Discretization of the control equations

Energy conservation is the physical basis for solving heat conduction problems. The integral and differential forms of the heat conduction equation are analyzed below [17]. The differential form finite difference scheme is calculated through the following steps:

(1) Mass conservation

The mass conservation equation for the gas phase is expressed as follows:


$$\frac{\rho_{g}^{j}V_{g}^{j+1}-\rho_{g}^{j}V_{g}^{j}}{\Delta \tau }+\frac{\rho_{g}^{j+1}V_{g}^{j}-\rho_{g}^{j}V_{g}^{j}}{\Delta \tau }=m_{v}^{j}$$ (7)

The mass conservation equation for the liquid phase is expressed as follows:


$$\frac{\rho_{l}^{\;j}V_{l}^{\;j+1}-\rho_{l}^{\;j}V_{l}^{\;j}}{\Delta \tau }+\frac{\rho_{l}^{\;j+1}V_{l}^{\;j}-\rho_{l}^{\;j}V_{l}^{\;j}}{\Delta \tau }=-m_{v}^{\;j}$$ (8)

(2) Energy conservation

The energy conservation equation for the gas phase is expressed as follows:


$$\rho_{g}^{\;j}V_{g}^{\;j}\frac{h_{g}^{\;j+1}-h_{g}^{\;j}}{\Delta \tau }+m_{v}^{\;j}h_{g}^{\;j}=\alpha_{bg}^{\;j}F_{bg}^{\;j}\left(T_{b}^{\;j}-T_{g}^{\;j}\right)-\alpha_{sg}^{\;j}F_{s}^{\;j}\left(T_{g}^{\;j}-T_{s}^{\;j}\right)+m_{v}^{\;j}{\left(h_{s}^{g}\right)}^{\;j}$$ (9)

The energy conservation equation for the liquid phase at the gas-liquid interface is expressed as follows:


$$\rho_{l}^{j}V_{l}^{j}\frac{h_{l}^{j+1}-h_{l}^{j}}{\Delta \tau }-m_{v}^{j}h_{l}^{j}=\alpha_{bl}^{j}F_{bl}^{j}\left(T_{b}^{j}-T_{l}^{j}\right)-\alpha_{sl}^{j}F_{s}^{j}\left(T_{l}^{j}-T_{s}^{j}\right)+m_{v}^{j}{\left(h_{s}^{l}\right)}^{j}$$ (10)

(3) The evaporation calculation is expressed as follows:

$$m_{v}^{\;j}=\frac{\alpha_{sg}^{\;j}F_{s}^{\;j}\left(T_{g}^{\;j}-T_{s}^{\;j}\right)-\alpha_{sl}^{\;j}F_{s}^{\;j}\left(T_{l}^{\;j}-T_{s}^{\;j}\right)}{\gamma_{s}^{\;j}}$$ (11)

(4) The volume conservation equation is expressed as follows:

$$V=V_{g}^{\;j+1}+V_{l}^{\;j+1}$$ (12)

(5) The heat transfer equation is expressed as follows:

$$Q=\frac{\alpha_{bl}\alpha_{hw}\lambda_{e}F_{bl}F_{w}F_{b}\left(T_{hw}-T_{l}\right)+\alpha_{bg}\alpha_{hw}\lambda_{e}F_{bg}F_{w}F_{b}\left(T_{hw}-T_{g}\right)}{\lambda_{e}\alpha_{hw}F_{w}F_{b}+\alpha_{bl}F_{bl}\left(\sigma \alpha_{h}F_{w}+\lambda_{e}F_{b}\right)+\alpha_{bg}F_{bg}\left(\sigma \alpha_{h}F_{w}+\lambda_{e}F_{b}\right)}$$ (13)

The parameter calculation equations [18] are expressed as follows:


$$P^{j+1}=133.3\times 10^{\left[A_{i}+B_{i}/T_{s}^{j+1}+C_{i}\log_{10}T_{s}^{j+1}+D_{i}T_{s}^{j+1}+E_{i}{\left(T_{s}^{j+1}\right)}^{2}\right]}$$ (14)

where the terms *A*, *B*, *C*, *D*, and *E* are obtained from reference; *P* is the pressure inside the storage tank, measured in Pascals (Pa).


$$\begin{array}{cc} h_{g}^{\;j+1}-h_{g}^{\;j} & =\{A_{i}\left[\left(T_{g}^{\;j+1}\right)-\left(T_{g}^{\;j}\right)\right]+\frac{B_{i}}{2}\left[{\left(T_{g}^{\;j+1}\right)}^{2}-{\left(T_{g}^{\;j}\right)}^{2}\right]+\frac{C_{i}}{3}\left[{\left(T_{g}^{\;j+1}\right)}^{3}-{\left(T_{g}^{\;j}\right)}^{3}\right]+\frac{D_{i}}{4}\left[{\left(T_{g}^{\;j+1}\right)}^{4}-{\left(T_{g}^{\;j}\right)}^{4}\right]\\ & \;+\frac{E_{i}}{5}\left[{\left(T_{g}^{\;j+1}\right)}^{5}-{\left(T_{g}^{\;j}\right)}^{5}\right]\} \end{array}$$ (15)

where the terms *T*_*ci*_ represent the critical temperature of the components, and *A* and *B* are data obtained from Tables 5-1 and 5-2 in reference.


$$h_{l}^{j+1}-h_{l}^{j}=\left\{A_{i}\left[\left(T_{l}^{j+1}\right)-\left(T_{l}^{j}\right)\right]+\frac{B_{i}}{2}\left[{\left(T_{l}^{j+1}\right)}^{2}-{\left(T_{l}^{j}\right)}^{2}\right]+\frac{C_{i}}{3}\left[{\left(T_{l}^{j+1}\right)}^{3}-{\left(T_{l}^{j}\right)}^{3}\right]+\frac{D_{i}}{4}\left[{\left(T_{l}^{j+1}\right)}^{4}-{\left(T_{l}^{j}\right)}^{4}\right]\right\}$$ (16)

$$\rho_{l}^{j+1}=A_{i}\cdot B^{-{\left(1-T/T_{c}\right)}^{n}}$$ (17)

where the terms *A*, *B*, *C*, *D*, and *E* are data obtained from Tables 3-1 and 3-2 in reference [19]; *h* represents the specific enthalpy of the liquid, measured in J/kg.


$$\rho_{s}^{j+1}=A_{i}\cdot {B_{i}}^{-{\left(1-T_{s}/T_{c}\right)}^{n}}$$ (18)

$$\gamma^{j+1}=\frac{10^{6}}{M_{i}}\cdot A_{i}\cdot {\left(1-T_{s}^{j}/T_{ci}\right)}^{n}$$ (19)

where the terms *A*, *n*, and *T*_*c*_ are data obtained from Tables 8-1 and 8-2 in reference. *γ* represents the latent heat of evaporation at the interface, measured in J/kg.


$$\begin{array}{cc} {\left(h_{s}^{l}\right)}^{\;j+1}-{\left(h_{s}^{l}\right)}^{\;j} & =\{A_{i}\left[\left(T_{s}^{\;j+1}\right)-\left(T_{s}^{\;j}\right)\right]+\frac{B_{i}}{2}\left[{\left(T_{s}^{\;j+1}\right)}^{2}-{\left(T_{s}^{\;j}\right)}^{2}\right]+\frac{C_{i}}{3}\left[{\left(T_{s}^{\;j+1}\right)}^{3}-{\left(T_{s}^{\;j}\right)}^{3}\right]\\ & \;+\frac{D_{i}}{4}\left[{\left(T_{s}^{\;j+1}\right)}^{4}-{\left(T_{s}^{\;j}\right)}^{4}\right]+\frac{E_{i}}{5}\left[{\left(T_{s}^{\;j+1}\right)}^{5}-{\left(T_{s}^{\;j}\right)}^{5}\right]\} \end{array}$$ (20)

$${\left(h_{s}^{g}\right)}^{j+1}={\left(h_{s}^{l}\right)}^{j+1}+\gamma_{s}^{j+1}$$ (21)

Initial conditions: ${T_{g}}^{1}={T_{l}}^{1}={T_{s}}^{1};V_{total}=V_{g}+V_{l};hg^{1}=400.38\;\text{kJ/kg; }{h_{l}}^{1}=916.74\;\;\text{kJ/kg}$.

## 5 Calculation program

**Fig 3:**
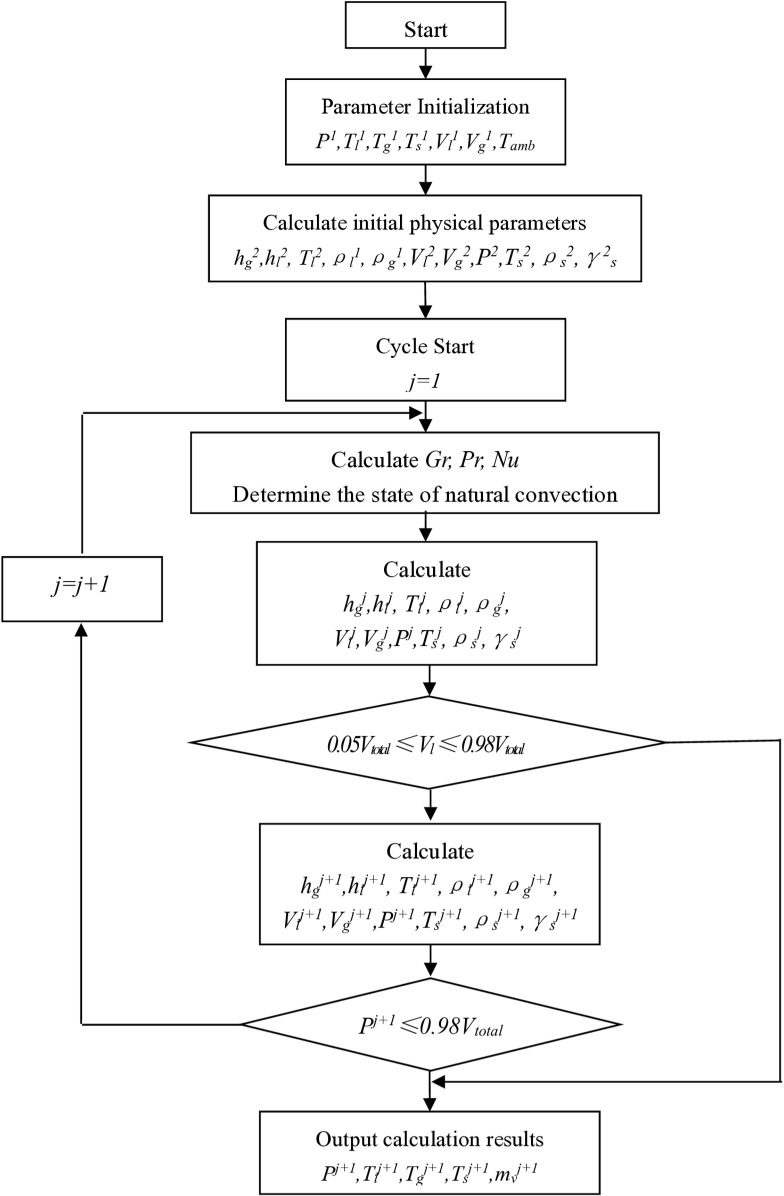
Pressure calculation flow chart of LNG rail container.

Based on the above calculation model and discrete equations, a calculation software has been developed. The known parameters that need to be input into the software include: total volume of the storage tank, inner diameter, outer diameter, length of the cylindrical section of the tank body, thickness of the insulation layer, thermal conductivity, initial pressure inside the tank, initial filling rate inside the tank, initial gas phase temperature inside the tank, initial liquid phase temperature inside the tank, initial molar ratio of evaporated gas inside the tank, initial molar ratio of LNG liquid components, ambient temperature, etc. During the calculation process, the storage tank is always in a sealed state. The calculation constraints are: pressure inside the storage tank P ≤ 3 MPa, filling rate 0.030 ≤ φf ≤ 0.90. The calculation steps are shown in Fig 3.

Model calculation assumptions:

(1) At the initial state, the temperatures of the inner tank, support components, and LNG components are the same.
(2) The temperature field inside the entire storage tank is divided into three sections: liquid phase temperature field, gas-liquid interface temperature field, and gas phase temperature field. The thickness of the gas-liquid interface is negligible, serving only as a transition zone where the liquid phase transforms into the gas phase, and the gas-liquid interface is always in a saturated state. The saturated pressure corresponding to the saturated temperature is the pressure inside the storage tank.
(3) During the evaporation process, the temperature fields of the gas phase and liquid phase of the LNG components inside the storage tank are considered uniform temperature fields.

Evaporation rate is an important parameter for assessing the sealed performance of LNG storage tanks, and it is also a crucial parameter for design. In engineering, it is generally assumed that the temperatures of the gas phase and liquid phase are always the same, and the liquid phase is always in a saturated state. However, due to different evaporation rates of each component, the molar fractions of each component in the gas phase and liquid phase differ. As the pressure inside the tank increases, the boiling point of LNG also changes accordingly. Therefore, to meet design requirements, the evaporation rate of the storage tank must be calculated and determined.

## 6 Numerical calculation of the evaporation process for railway LNG tank containers

The selected railway LNG tank container is designed and manufactured by Shijiazhuang Enric Gas Equipment Co., Ltd. The LNG tank container consists of a tank and an outer frame. The tank includes five parts: inner tank, outer tank, inner and outer tank support structures, insulation materials, and safety accessories. The connection between the tank and the outer frame uses a welded structure, with the safety accessories and valves positioned at one end of the tank. The technical parameters and units of the test railway LNG tank container is shown in Table 1.

**Table 1.** Model of LNG rail container.

Parameters/Units	Values
Outer Tank Dimensions	22412mm11866mm
Inner Tank Dimensions/mm	222611475
Inner Tank Geometric Volume/m³	42.5
Tank Design Thickness (Cylindrical Section)	7.53mm (Inner),4.25mm (Outer)
Tank Design Thickness (End Cap)	7.51mm(Inner)，5.92mm（Outer）
Design Temperature/°C	Inner Tank -196；Outer Tank -45
Tank Material	Inner Tank S30408, Outer Tank 16MnDR
Frame Material	Q345E
Valve and Piping Material	0Cr18Ni9
Insulation Method	High Vacuum Multilayer Insulation Wrapping
Inter-Tank Pressure/Pa	5.0 × 10^-3^
Tank Container Operating Pressure/MPa	0.63
Tank Container Design Pressure/MPa	0.66
Safety Valve Opening Pressure/MPa	0.70
Safety Valve Reseating Pressure/MPa	0.63
Static Daily Evaporation Rate/(%/day)	LNG 0.19, Liquid Nitrogen 0.26
Empty Weight/kg	13980
Tank Container Rated Gross Weight/kg	30480
Insulation Method	High Vacuum Multilayer Insulation Wrapping

(1) Formulas for calculating total volume and surface area

Total volume is expressed as follows:


$$V=\frac{\pi }{4}D^{2}\cdot L+2\cdot \frac{D^{3}}{24}\pi$$ (22)

The lateral surface area of a half-ellipsoid is expressed as follows:


$$S_{1}=\iint_{D}\sqrt{1+{\left(\frac{\partial z}{\partial x}\right)}^{2}+{\left(\frac{\partial z}{\partial y}\right)}^{2}}dxdy\approx 1.3802\cdot \frac{D^{2}}{4}\pi$$ (23)

Total surface area is expressed as follows:


$$S=\pi DL+1.3802\pi \cdot \frac{D^{2}}{2}$$ (24)

(2) Parameter calculation for liquid level *h*
(1) Volume calculation

The volume of the ellipsoid part is expressed as follows:


$$V_{1}(h)=\frac{\pi }{4}\cdot h^{2}\left(\frac{D}{2}-\frac{h}{3}\right)$$ (25)

The volume of the main sector is expressed as follows:


$$V_{2}(h)=\frac{D^{2}L}{4}\cdot \arccos(1-2h/D)-\sqrt{Dh-h^{2}}\cdot (\frac{D}{2}-h)\cdot L$$ (26)

Total volume is expressed as follows:


$$V_{2}(h)=\frac{D^{2}L}{4}\cdot \arccos(1-2h/D)-\sqrt{Dh-h^{2}}\cdot (\frac{D}{2}-h)\cdot L+\frac{\pi }{2}\cdot h^{2}(\frac{D}{2}-\frac{h}{3})$$ (27)

(2) Area calculation S:

The surface area of a half-ellipsoid is expressed as follows:


$$S_{1}=\iint_{D}\sqrt{1+{\left(\frac{\partial z}{\partial x}\right)}^{2}+{\left(\frac{\partial z}{\partial y}\right)}^{2}}dxdy=\int_{0}^{\theta }d\theta \int_{0}^{\sqrt{{\left(\frac{D}{2}\right)}^{2}-{\left(\frac{D/2-h}{\cos\theta }\right)}^{2}}}\sqrt{\frac{D^{2}}{4}+3t^{2}}dt$$ (28)

$$S(h)=0.5D\cdot L\cdot \arccos(1-2h/D)+2S_{1}(h)$$ (29)

$$F(h)=\pi \cdot \frac{1}{2}\left(Dh-h^{2}\right)+2\sqrt{Dh-h^{2}}\cdot L$$ (30)

$$P(h)=2l+1.027976\pi \cdot \frac{3}{2}\sqrt{Dh-h^{2}}$$ (31)

(3) Data analysis of non-destructive storage for railway LNG tank containers:

Basic calculation parameters are shown in Table 2:

**Table 2.** Components of LNG.

Components	Mole Fraction mol%
Methane	98.26
Ethane	0.941
Propane	0.178
Butane	0.0184
Nitrogen	0.6026

On August 13, 2013, at 15:35, the pre-cooling and filling of the LNG tank container began, and the filling ended on the 14th at 13:05. The filling records are shown in Table 3 for the first filling and Table 4 for the second filling, with details as follows.

**Table 3.** First fill recorder.

Container Number	Container #1	Container #2	Container #3	Container #4	Container #5
LNG Filling Weight (kg)	14960	14980	14960	14860	14920
LNG Filling Volume (m³)	35.04	35.08	35.04	34.80	34.94
Pressure After Filling (MPa)	0.05	0.03	0.05	0.02	0.03
Liquid Level (mmH2O)	750	740	730	740	730

**Table 4.** Second fill recorder.

Container Number	Container #1	Container #2	Container #3	Container #4	Container #5
LNG Filling Weight (kg)	14980	14920	14860	14940	14920
LNG Filling Volume (m³)	35.08	34.94	34.80	34.99	34.94
Pressure After Filling (MPa)	0.02	0.02	0.02	0.02	0.02
Liquid Level (mmH2O)	750	750	760	750	730

Test Method: Explosion-proof wireless digital pressure sensors (model SM39-DW-CP), wireless antennas, wireless transmission modules, and dedicated escort computer monitoring systems are used to monitor the pressure inside the tank container. Additionally, pressure gauge data are manually read when the vehicle is stationary.

(4) Comparison and Analysis of Experimental Data and Calculated Model Data. As shown in Table 5, the pressure changes values for LNG tank containers 1-5 compared with the calculated simulation values for container 6 show good consistency.

**Table 5.** Pressure difference of LNG rail containers.

Container Number	Container #1	Container #2	Container #3	Container #4	Container #5	Calculated value
The first outbound test maximum difference (MPa)	0.019	0.011	0.008	0.014	0.013	0.015
The first return test maximum difference (MPa)	0.006	0.005	0.014	0.011	0.016	0.012
The second outbound test maximum difference (MPa)	0.016	0.017	0.018	0.012	0.012	0.015
The second return test maximum difference (MPa)	0.006	0.006	0.012	0.006	0.016	0.012

**Fig 4.**
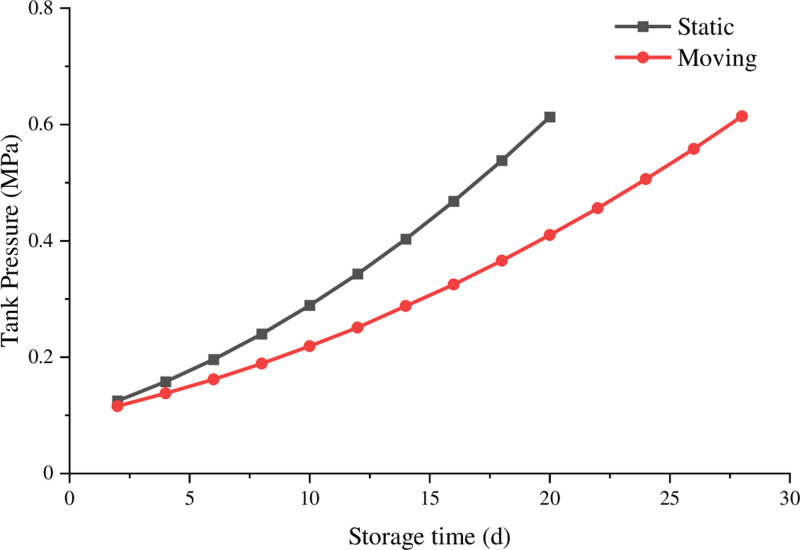
Influence of stillness and movement on LNG rail container storage time and pressure.

As shown in Fig 4., the pressure change values under vibration conditions within a unit of time are smaller than those under static conditions for the LNG tank container.

## 7 Comparison of calculation model and experimental data

To ensure experimental safety, this paper uses experimental data on the storage characteristics of low-temperature liquid nitrogen tanks to validate the calculation model. The main experimental equipment is a horizontal liquid nitrogen storage tank, with basic parameters shown in Table 6 [20]:

**Tab 6.** Parameters of horizontal nitrogen tank.

Parameters/Units	Values
Inner Tank Working Pressure/ MPa	3.5
Inner Tank Design Pressure/ MPa	4
Inner Tank Working Temperature/ °C	-196
Inner Tank Design Temperature/ °C	-196
Inner Tank Volume/ m³	2.22
Filling Rate/ (%)	90
Inner Tank Pressure Test Pressure	4.6MPa
Interlayer Static Leak Rate	2 × 10^-4^Pa/s
Daily Evaporation Rate	≤0.7%/d
Inner Tank Main Dimensions	1565×Φ1200
Outer Tank Working Pressure/ 1 MPa	-0.
Outer Tank Design Pressure/ MPa	-0.1
Outer Tank Working Temperature	Room Temperature
Outer Tank Design Temperature	Room Temperature
Interlayer Volume/ m³	2.11
Interlayer Sealing Vacuum Degree/ Pa	≤5 × 10^-3^
Outer Tank Working Medium	High Vacuum Multilayer
Insulation Type	High Vacuum Multilayer Insulation
Inner Tank Working Medium	Liquid Nitrogen
Equipment Overall Dimensions	3480×φ1420 × 1660

Before starting the experiment, the tank was kept stationary for more than 24 hours as required to ensure that all media inside the tank were at the same temperature condition, with an initial tank pressure of 1 atmosphere. The experiment was conducted four times, and the relationship between pressure changes over time during the experiment is shown in Table 7.

**Table 7.** Experimental data.

Serial Number	Test Time (h)	Initial Liquid Level (m)	Initial Pressure (MPa)	Initial Filling Rate	Final Pressure (MPa)
1	288	0.752	0.1	80%	3.00
2	197	0.436	0.1	39%	3.06
3	280	0.837	0.1	89%	2.90
4	263	0.600	0.1	63%	3.12

**Fig 5.**
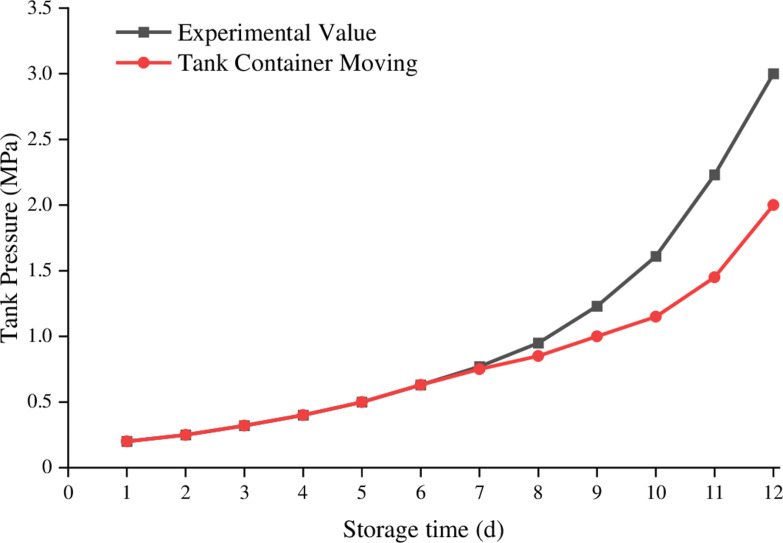
Compare filling 80% liquid nitrogen experimental data with calculated value.

Using the established model, simulations were conducted on a 2m³ low-temperature liquid nitrogen storage tank based on the initial experimental conditions. The initial calculation pressure of the storage tank was 0.1 MPa, and the experimental termination pressure was 3 MPa. Simulations were conducted under three experimental conditions with initial filling rates of 80%, 63%, and 39%. The comparison between the simulated values and actual experimental values at an 80% liquid nitrogen filling rate is shown in Fig 5.

**Fig 6.**
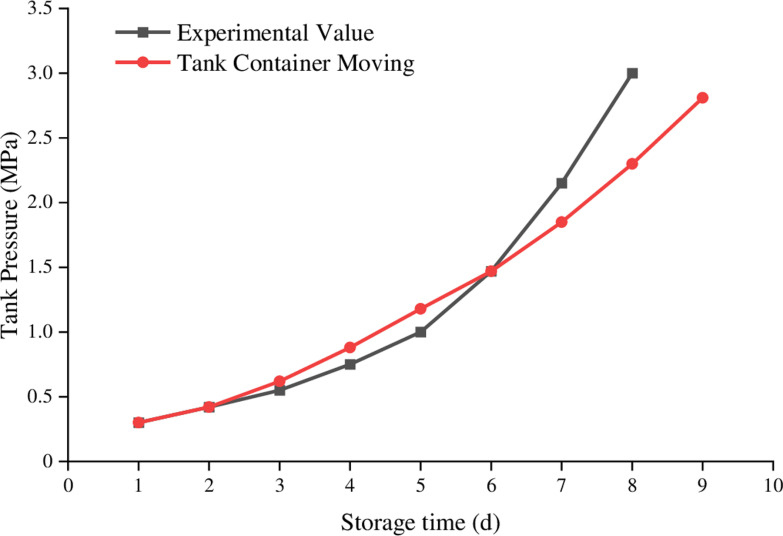
Compare filling 63% liquid nitrogen experimental data with calculated value.

The comparison between the simulated values and actual experimental values at a 63% liquid nitrogen filling rate is shown in Fig 6.

**Fig 7.**
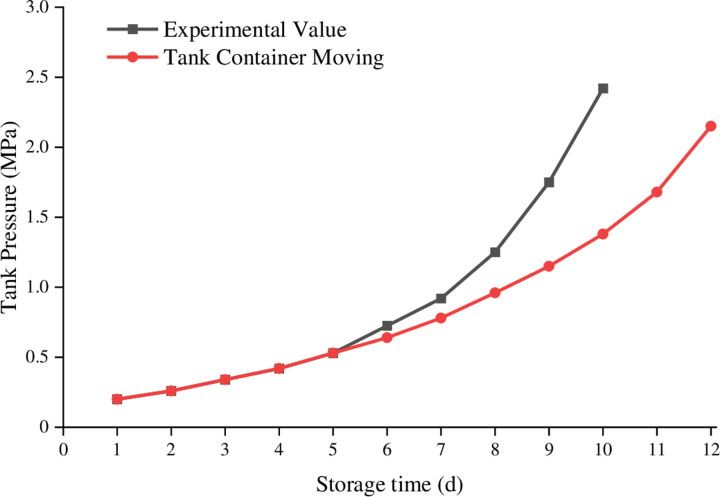
Compare filling 39% liquid nitrogen experimental data with calculated value.

The comparison between the simulated values and actual experimental values at a 39% liquid nitrogen filling rate is shown in Fig 7.

From the above comparison analysis, it can be concluded that in the early stages of storage, the model’s simulated values closely match the actual experimental values without significant deviations. However, in the later stages of storage, there are deviations between the model’s simulated values and the actual experimental values, with larger deviations occurring at medium filling rates.

The internal gas pressure of the tank increases with time at different initial filling rates, and there is an “optimal initial filling rate value” for the tank. Within the safe pressure range, as the filling rate increases, the safe storage time of the tank first increases and then decreases. The optimal filling rate is generally between 0.82 and 0.9.

## 8 Conclusion

The vibration-induced three-phase evaporation calculation model for railway tank containers transporting LNG proposed in this study has been established and experimentally validated. The experimental results indicate that this control method can account for factors affecting the pressure and evaporation rate of sealed LNG storage tanks. The concludes can be drawn as follows.

(1) A three-phase evaporation calculation model for LNG tank container transportation was established and compared with the pressure variations observed during the Golmud-Lhasa section tests of railway LNG tank containers, showing good agreement.
(2) Using the established model, simulations were conducted on a 2m³ low-temperature liquid nitrogen storage tank under the initial experimental conditions. Within the safe pressure range, as the filling rate increases, the safe storage time of the tank first increases and then decreases. The optimal filling rate is generally between 0.82 and 0.9.

## Supporting information

S1 Dataset The Figure 4 to Figure 7 (ZIP)
